# Supplementary material for: Complexome profiling of the Chlamydomonas psb28 mutant reveals TEF5 as an early PSII assembly factor
Source: Plant Cell. 2025 Mar 17;37(6):koaf055. doi: 10.1093/plcell/koaf055 (PMC12164590; doi:10.1093/plcell/koaf055)
Supplement: koaf055_Supplementary_Data [file koaf055_supplementary_data.zip › psb28_interactive.zip!/plots/intensity/qualityPlots/peptides/CGL73_Cre12.g510850.t1.2.html]

### Cre12.g510850.t1.2

Trivial name: CGL73  
SeqCoverage [median]: 11.80  
Mass: 30.72 kDa  
  
 

| Sequence | MissedCleavage | Mass [Da] | wtIntensity | muIntensity | Score |
| --- | --- | --- | --- | --- | --- |
| DAAFEEVQDAR | 0 | 1249.56 | 0.00 | 1273.84 | 96.89 |
| EAIELAFDR | 0 | 1062.53 | 0.00 | 857.27 | 72.48 |
| KVNDLIDPTITTR | 1 | 1484.82 | 700.12 | 1396.28 | 45.12 |
|  |  |  | 700.1 | 3527.4 |  |
